# Physics-Guided Gaussian Process Mapping of Strong-Gradient Radiation Fields from Mobile Robot Surveys: The Role of Sampling Geometry

**DOI:** 10.3390/s26185697

**Published:** 2026-09-08

**Authors:** Hui Li, Qing Fan, Liye Liu, Hua Li, Faguo Chen, Mingming Wang, Deyuan Li, Yuan Zhao, Zhi Chen

**Affiliations:** 1 School of Nuclear Science and Technology, University of Science and Technology of China, Hefei 230026, China; huberylee@mail.ustc.edu.cn; 2 CNNC Key Laboratory of Radiation Protection Technology, Taiyuan 030006, Chinazhaoyuan@cirp.org.cn (Y.Z.); 3 China Institute for Radiation Protection, Taiyuan 030006, China

**Keywords:** radiation mapping, mobile robot, Gaussian process regression, physics-informed machine learning, spatial interpolation, uncertainty quantification

## Abstract

Radiation fields around collimated or shielded sources exhibit strong gradients whose accurate delineation is critical for worker protection and emergency response. Mobile robots can survey such fields, but they sample sparsely and irregularly along their trajectories, and it remains unclear which reconstruction method can be trusted, and where. Using a single dominant collimated source in a two-dimensional indoor setting, this study shows that the answer depends decisively on sampling geometry, and proposes a physics-guided Gaussian process (GP) that performs reliably under trajectory-constrained sampling. A tracked robot combining light detection and ranging (LiDAR)-based simultaneous localization and mapping (SLAM) with a γ dose-rate detector surveyed a collimated Cs-137 field in seven independent runs, and all methods were evaluated under both random hold-out (interpolation near visited locations) and spatial block cross-validation (extrapolation into unvisited regions); truth-referenced evaluation against a dense reference field is provided by Poisson-sampled simulations, while experimental accuracy is cross-validated on held-out measurements. Under uniform sampling, a multilayer perceptron (MLP) robustly outperformed GP variants ($R^{2}=0.95$, stable across 18 seed combinations); under trajectory sampling, its advantage vanished at visited locations and reversed catastrophically in unvisited regions. The proposed physics-guided GP, which uses a fitted collimated-beam template as the GP mean with a Matérn 3/2 residual process, achieved the highest extrapolation $R^{2}$ (median 0.61; best baseline 0.31), reduced the extrapolation error by 32–69% relative to all eight baselines, recovered interpretable source parameters, and provided predictive intervals with approximately calibrated region-level coverage (point-wise error ranking remains weak); a runtime fit-quality gate further renders the correctness of the embedded prior an observable quantity, so the method flags when its own assumptions fail. These results offer quantitative guidance for method selection in robotic radiation mapping under the as low as reasonably achievable (ALARA) principle.

## 1. Introduction

Characterizing dose-rate distributions inside nuclear facilities underpins routine operation, decommissioning, and emergency response [1,2]. Mobile robots are increasingly deployed to acquire such measurements in place of human surveyors, following the as low as reasonably achievable (ALARA) principle [3,4,5,6]. The surveys conducted after the Fukushima Daiichi accident remain the canonical example [1]. The deployment envelope has since broadened to aerial platforms surveying open terrain [7] and to multi-robot teams searching cooperatively during emergency response [8], while imaging modalities such as Compton cameras characterize source terms directly for decommissioning [9]. A robotic survey, however, does not produce a dose map directly: it produces sparse, noisy point measurements distributed irregularly along the vehicle trajectory, from which a continuous field must be reconstructed [10,11,12].

The reconstruction problem is hardest precisely where it matters most. Radiation fields near collimated openings, shielding gaps, and maze entrances exhibit abrupt, strongly anisotropic transitions in which dose rate changes by orders of magnitude over tens of centimeters. Misplacing such a boundary in either direction carries asymmetric costs: an outward error wastes access time, while an inward error exposes workers. A reconstruction that draws a confident but wrong boundary is therefore more dangerous than one that draws an honest, uncertainty-qualified boundary. As mapping outputs are increasingly consumed by downstream planners rather than human experts [13,14], the ability of a method to report *where it should not be trusted* becomes as important as its point accuracy.

Two methodological traditions dominate robotic radiation mapping. The geostatistical tradition, comprising kriging and Gaussian process regression (GPR), handles irregular, noisy, clustered data naturally and supplies predictive variance [15,16,17]. West et al. demonstrated GPR mapping of an operating reactor with a mobile robot [15]. Jung et al. adapted the inverse-square law into a GPR kernel for three-dimensional mapping [16]. Zhang et al. introduced multi-kernel weighting to accommodate obstructions [18]. Poisson kriging addresses counting statistics explicitly [19]. These methods, however, typically employ isotropic, stationary priors whose smoothness assumptions blur exactly the abrupt boundaries that strong-gradient scenarios present. The machine-learning tradition, including backpropagation networks [20], U-Net variants [21], physics-informed networks that embed transport physics in the loss [22], and coordinate-input multilayer perceptrons (MLPs), imposes no smoothness prior and can, in principle, represent sharp transitions, but provides no uncertainty output, and its dependence on the quantity and spatial distribution of training samples is rarely examined. Reported results are seldom accompanied by initialization-seed statistics.

A further gap concerns evaluation. Robotic survey data are spatially autocorrelated along trajectories, so the ubiquitous random train/test split largely measures interpolation between immediately adjacent points and can substantially overstate performance in unvisited regions, a pitfall documented across spatial prediction domains [23]. Few studies of robotic radiation mapping have systematically compared reconstruction methods under both a random hold-out protocol and a spatially blocked protocol, and the calibration of GP uncertainty under the latter has rarely been assessed.

This paper addresses these gaps with three contributions. First, using seven independent robotic surveys of a collimated Cs-137 field and Poisson-noise simulations, we show that the ranking of reconstruction methods is determined by sampling geometry: under uniform sampling, the MLP is robustly superior at recovering abrupt boundaries, whereas under trajectory sampling, its advantage disappears at visited locations and reverses severely in unvisited regions. Second, we propose a physics-guided GP for strong-gradient fields, in which a parametric collimated-beam template is fitted as the GP mean function and a Matérn 3/2 process models the residual, and show that it achieves the highest extrapolation $R^{2}$ among eight baselines (median 0.61 versus at most 0.31), while independently recovering source position, beam direction, and cone half-angle, consistent with the actual apparatus in six of seven runs (the exception being a known mirror-direction degeneracy that leaves the position and half-angle unaffected). A runtime fit-quality gate additionally makes the correctness of this prior an observable quantity, so the method can flag when its own assumptions break down. Third, we report the calibration of the GP predictive uncertainty under spatial block cross-validation, demonstrating that the resulting maps convey usable confidence information for survey planning and ALARA decision support. In relation to the GPR line of work [15,16,18], our approach extends the physics-informed direction [24] from isotropic inverse-square priors to anisotropic, collimation-aware priors; in relation to neural approaches [20,21], our results delimit the sampling conditions under which they can be recommended.

## 2. Materials and Methods

### 2.1. Robotic Survey Platform and Data Acquisition

**Figure 1:**
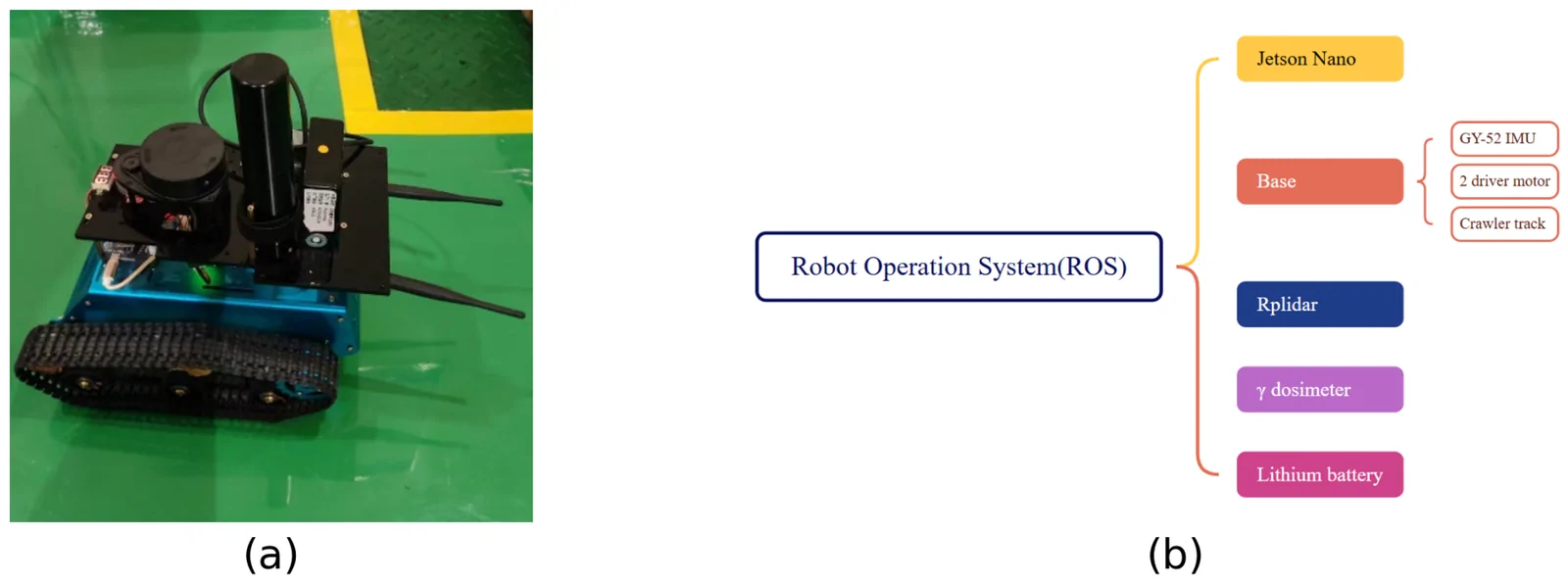
Mobile survey platform: (**a**) photograph of the tracked robot; (**b**) ROS-based system architecture, comprising the Jetson Nano computing unit, the tracked base with GY-52 IMU and two drive motors, the RPLIDAR sensor, the γ dosimeter, and the lithium battery.

The survey platform (Figure 1) is a tracked mobile robot composed of an onboard computing unit, a tracked chassis, a light detection and ranging (LiDAR) sensor, a γ dose-rate detector, and a lithium battery power system. The tracked chassis measures 309.8 mm in length, 237.2 mm in width, and 361.1 mm in height. The reconstruction methodology itself is agnostic to the platform dimensions: the footprint matters only insofar as it sets the minimum standoff from walls and obstacles, and thereby bounds the region the trajectory can reach, while the detector mounting height defines the horizontal plane on which the two-dimensional dose map is reconstructed. The Robot Operating System (ROS Noetic) [25] serves as the software framework, providing unified management and communication scheduling for all sensors and actuators. The platform is teleoperated over Wi-Fi and sustains more than 2 h of continuous operation, sufficient for indoor facility inspection tasks.

The computing unit is an NVIDIA Jetson Nano (NVIDIA Corporation, Santa Clara, CA, USA) edge module with a 128-core GPU and a quad-core ARM Cortex-A57 CPU, which provides adequate processing capacity at low power for sensor fusion, simultaneous localization and mapping (SLAM), and real-time processing of radiation data. A Slamtec RPLIDAR (Slamtec Co., Ltd., Shanghai, China) sensor provides 360° range measurements for two-dimensional environment mapping. The inertial measurement unit (IMU) is a GY-52 module integrating a three-axis gyroscope, a three-axis accelerometer, and a three-axis magnetometer for attitude and motion estimation. The γ dose-rate detector, which reports the ambient dose equivalent rate ${\dot{H}}^{*}\left(10\right)$ (referred to as “dose rate” hereafter), is mounted vertically on top of the platform, with the center of its sensitive volume 306.2 mm above the floor, which defines the height of the mapped plane. The rigid transform from the SLAM reference frame to the detector, a horizontal offset of (−41.1,−67.7) mm in the robot body frame, is registered in the robot’s URDF (Unified Robot Description Format) model, so that each dose-rate reading is georeferenced through the ROS tf tree to the detector’s own (x,y) position in the SLAM map rather than to the platform pose; the detector plane merely lies at a different height from the LiDAR scanning plane. It weighs less than 200 g, covers 0.1 µSv/h–10 Sv/h with an energy response of 60 keV–3 MeV and a response time below 3 s, and streams readings to the computing unit over USB [26]. Two-dimensional maps and poses are produced by the gmapping SLAM algorithm [27] fusing LiDAR, IMU, and wheel-odometry data. Dose-rate readings are logged at approximately 1 Hz, paired with the nearest-timestamp SLAM pose, and georeferenced to the detector position through the URDF transform described above.

**Figure 2:**
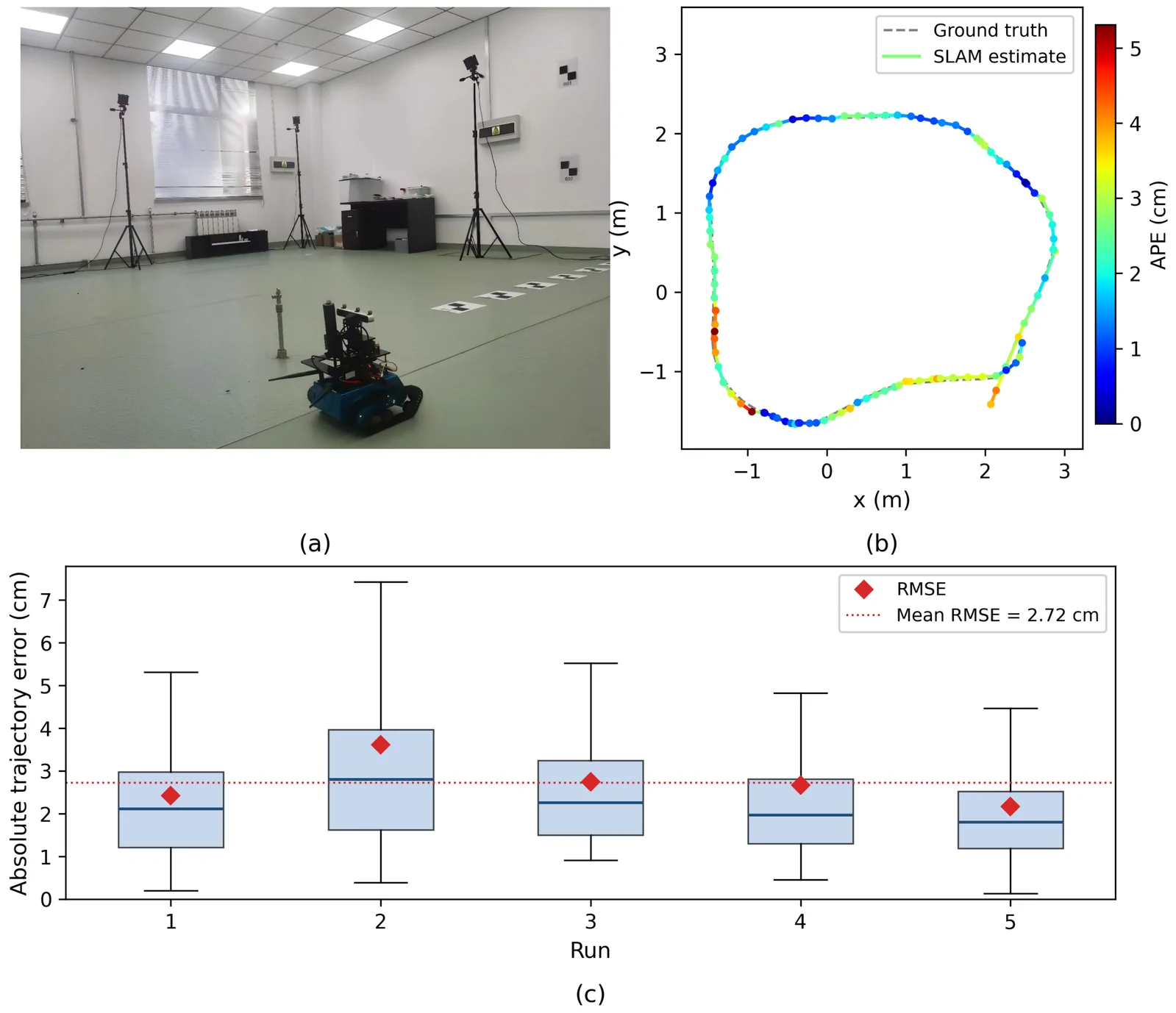
Localization accuracy evaluation: (**a**) the tracked robot, carrying reflective markers, in the motion-capture laboratory; (**b**) SLAM-estimated trajectory after SE(3) alignment, colored by the point-wise absolute pose error (APE), overlaid on motion-capture ground truth (dashed gray; run 1); (**c**) APE distribution over the five repeated localization–evaluation runs conducted in the motion-capture laboratory (independent of the seven radiation survey runs; boxes: interquartile range; diamonds: per-run RMSE; dotted line: mean RMSE 2.72±0.49 cm).

Localization accuracy was evaluated against an eight-camera NOKOV (NOKOV Science and Technology Co., Ltd., Beijing, China) motion-capture system (3D accuracy ±0.15 mm) as ground truth. After SE(3) alignment by the Umeyama method [28], the absolute trajectory error (ATE) over five repeated runs had a root-mean-square error (RMSE) of 2.72±0.49 cm (Figure 2), adequate for dose mapping at the decimeter scale. This localization-accuracy evaluation is a separate experiment conducted in the motion-capture laboratory, where trajectory ground truth is available; its five repetitions are therefore unrelated to, and need not match, the seven radiation survey runs described below. The median survey speed across the seven radiation runs was 0.14 m/s (max ≤ 0.3 m/s). Combined with the detector response time, the spatial smearing of an individual reading is approximately 0.4 m, below the characteristic structure scale of the fields considered here.

**Figure 3:**
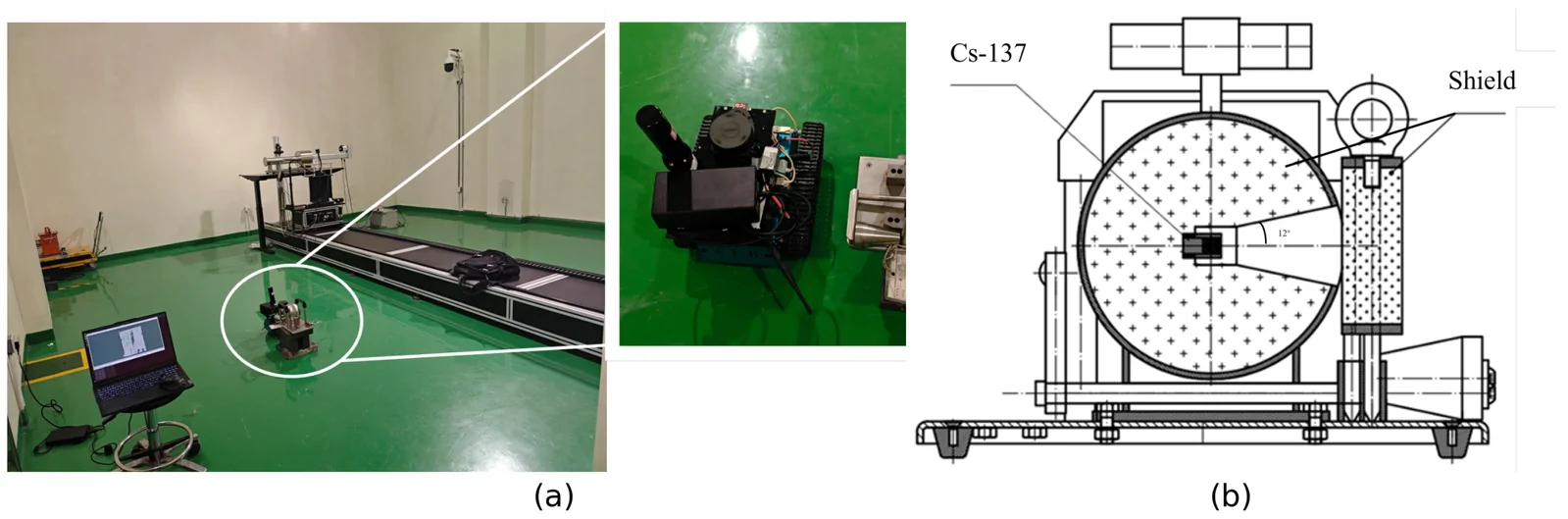
Collimated Cs-137 source used in the radiation experiments: (**a**) experimental setup; (**b**) structure of the source and its shielding. The source has a nominal activity of $7.4\times 10^{8}$ Bq (0.74 GBq) and a collimator half-angle of 12°.

Radiation experiments were conducted in a laboratory of a national secondary calibration facility. A collimated Cs-137 source (Figure 3; lead collimator, fan-shaped aperture with nominal half-angle of 12°; activity $7.4\times 10^{8}$ Bq, i.e., 0.74 GBq) was oriented toward a wall more than 3 m away. In each of seven independent runs, the operator teleoperated the robot around the room perimeter to build the map, then swept repeatedly across the beam region, yielding 521–2325 georeferenced dose-rate measurements per run after removing pre-convergence poses. All dose rates *d* (in µSv/h) were transformed as $y=\log_{10}\left(d+1\right)$, where the additive constant of 1 µSv/h keeps the transform finite at zero dose and sets the background floor. Readings below 1 µSv/h are thereby mapped to y≈0 and treated as background. The transform compresses the several-decade dynamic range of the collimated field into a numerically stable target while preserving the ordering of dose rates.

### 2.2. Physics-Guided Gaussian Process

We model the log-domain field as y(x)=m(x)+f(x)+ε, where m(x) is a parametric physics template, f(x)∼GP(0,k) is a residual Gaussian process with a Matérn 3/2 kernel plus white noise, and ε is observation noise. The template represents a single dominant collimated source:

(1) $$m\left(x\right)=\log_{10}\;\left(\frac{C\;g(\theta (x))}{r^{2}\left(x\right)}+b+1\right),$$

where *r* is the distance to the source position $\left(s_{x},s_{y}\right)$, softened as $r^{2}\to r^{2}+r_{c}^{2}$ with $r_{c}=0.3$ m to remove the inverse-square singularity in the near field, where the point-source assumption does not hold and measurements can hardly be taken; θ is the angular offset from the beam direction φ; and

(2) $$g\left(\theta \right)=l+\left(1-l\right)\;\sigma \;\left(\frac{\theta_{\mathrm{half}}-\theta }{w}\right)$$

is a sigmoid-smoothed sector profile with half-angle $\theta_{\mathrm{half}}$, transition width *w*, and leakage factor *ℓ*; *b* is the background level. The sigmoid replaces the ideal step-like sector edge with a differentiable transition, reflecting the finite penumbra of a physical collimator while keeping the template amenable to gradient-based least squares. Equation (1) is derived from the inverse-square law governing point-source radiation measurements [29], expressed in the logarithmic observation domain and modulated by the angular acceptance of the collimator. Equation (2) provides a smooth representation of that acceptance function. An inverse-square structure has previously been embedded in GP priors for radiation mapping in isotropic form [16]; Equations (1) and (2) extend this concept to the anisotropic, collimation-aware case. The eight parameters are estimated from training data by robust nonlinear least squares (soft-L1 loss) with multi-start initialization: the source position is initialized at the maximum reading, and the direction at the intensity-weighted bearing of the hottest 15% of points, with additional starts rotated by ±90° and 180° to escape directional local minima. GP hyperparameters (signal variance, length scale, noise variance) are estimated by maximizing the marginal likelihood [30] on a subsample of at most 800 points and then held fixed while conditioning on all training points.

The construction itself is a collimation-aware instantiation of a classical idea, namely GP regression with a parametric (non-zero) mean, known in geostatistics as universal or external-drift kriging [30], and in contemporary terms an instance of physics-informed machine learning [24]. It differs from network-based physics-informed approaches, which enforce the transport equation as a soft penalty during training [22], in that the physics enters as a closed-form prior mean and the residual retains an analytic posterior variance. The contribution here lies not in the construction but in the anisotropic template family, the dual-protocol evidence for when such a prior is necessary, and the misspecification analysis of Section 4.

Two design choices merit emphasis. First, the division of labor is deliberate: the template mean governs behavior far from data, where a zero-mean GP reverts to a constant and a coordinate-input neural network (such as the MLP baseline of Section 2.3) extrapolates without constraint, while the residual GP corrects local deviations near data and supplies predictive variance. Second, the method has a defined scope and a runtime adequacy check: it targets scenarios with a single dominant source of known family. Template adequacy is monitored by a quantitative fit-quality gate whose statistic is the normalized robust fit cost $\bar{c}$ (the soft-L1 objective of the template fit divided by the number of training points). Whenever $\bar{c}$ exceeds a threshold τ calibrated on correctly specified reference fits (τ=1.3× the largest $\bar{c}$ observed for correct templates on held-out surveys), the template is flagged as inadequate, triggering a runtime alarm that prompts supplementary measurement or manual review, with a conservative fallback to the plain GP (setting m(x) to a constant) as the default. Section 4 quantifies the gate’s detection and false-alarm rates. Multi-source and unknown-source-type scenarios are future work.

### 2.3. Baseline Methods

Eight baselines span the main traditions: (i) linear interpolation on the Delaunay triangulation with nearest-neighbor fill; (ii) plain GPR with Matérn 3/2 kernel plus constant (bias) kernel and white noise, hyperparameters by marginal likelihood, equivalent to ordinary kriging, i.e., a GP with unknown constant mean [30] (the configuration closest to prior robotic mapping work [15]); (iii) inverse-distance weighting (k=8, p=2); (iv) random forest regression (200 trees); (v) a coordinate-input MLP (64–32–16, tanh activation, limited-memory BFGS optimizer [31], L2 regularization α=0.01, standardized inputs), reported both as a single network and as a seed ensemble (two baselines); (vi) a multi-kernel weighted GP, a marginal-likelihood-fitted weighted sum of Matérn 3/2, Matérn 5/2, and RBF kernels at distinct length scales (with a bias term and white noise), instantiating the multi-kernel weighting proposed for obstructed fields [18]; and (vii) Poisson kriging, an ordinary-kriging GP whose per-point observation variance is set from Poisson counting statistics (proportional to the inverse local count; as the detector reports dose rate rather than raw counts, 1/(d+1) serves as the count proxy, clipped to [1/3,3] times its median to avoid a degenerate near-zero nugget) [19]. The clip range and nugget scale shift Poisson kriging’s absolute error by up to ∼0.1 MAE on the sparsest runs but leave it far behind the physics-guided GP under every setting tried (Protocol B MAE ≥0.32 vs. ≤0.20), so the comparison is insensitive to this heuristic. All methods are implemented in Python using scikit-learn (v1.3 or later) and SciPy (v1.10 or later) [32] (version requirements per the repository’s requirements.txt; https://github.com/Hubery-Lee/PhysicsGuidedGP-RadiationMapping, accessed on 16 August 2026). All methods receive identical training data and are evaluated on identical held-out points. No method is evaluated on an internal split.

### 2.4. Evaluation Protocols

**Simulation (truth-referenced).** A collimated Cs-137 source (activity $7.4\times 10^{8}$ Bq, detection efficiency 50%, leakage $10^{-4}$, half-angle 12°) defines the expected count rate at any location. Measured counts are drawn from a Poisson distribution, so that relative uncertainty varies naturally with position (≈0.02% at 1 m in-beam; ≈26% at the far out-of-beam regions), consistent with counting statistics [19,29]. The detection efficiency and leakage factor set the count level and the out-of-beam floor, respectively. The sampled counts are then log-transformed with the same $\log_{10}\left(\cdot +1\right)$ mapping used for the experimental dose rates, so simulation and experiment are scored on a common scale. Two hundred uniformly distributed samples are drawn per run, and six methods are compared: linear interpolation, GPR with a radial basis function (RBF) kernel, GPR with a Matérn 3/2 kernel plus bias, a multi-kernel weighted GP, Poisson kriging, and the MLP. Reconstructions are scored against the true field on a dense grid. The physics-guided GP is excluded from the simulation comparison because the simulated field is generated by the same collimated-beam model that its template embeds. Evaluating it here would test the method against its own generative assumptions and overstate its performance. It is therefore assessed only on the experimental data, where the template is at best an approximation to the real field. Robustness is assessed over 3 data seeds × 6 network seeds.

**Experiments (two protocols).** No independently measured dense reference radiation field was established for the physical experiments. Unlike in simulation, where the underlying field is known at every location, the true continuous radiation field in the laboratory is not directly observable; independent reference measurements would themselves provide only discrete samples with measurement uncertainty rather than a dense ground-truth field. Experimental performance is therefore assessed by cross-validation on held-out robot measurements, whereas the simulation permits direct truth-referenced evaluation against the known field. Because the held-out measurements are acquired by the same detector system, this protocol evaluates out-of-sample predictive performance but cannot reveal systematic errors shared by the measurement chain, such as energy-response, angular-response, or detector-mounting biases. Experimental results should therefore be interpreted as cross-validation performance rather than absolute dosimetric accuracy. Protocol A (random hold-out, 80/20) measures interpolation quality near visited locations. Protocol B (spatial block cross-validation) partitions each survey area into a 4×4 grid of blocks and performs group 5-fold cross-validation by block, measuring reconstruction in regions the trajectory never visited. Metrics are the mean absolute error (MAE), the RMSE, and the coefficient of determination ($R^{2}$) in the log domain, which capture average accuracy, large local errors (boundary failures), and spatial-structure recovery, respectively, plus a boundary F1 score: classification agreement, at held-out points, of in-beam status defined by a fixed threshold one decade above background (10 µSv/h). Method differences over the seven runs are tested by two-sided paired Wilcoxon signed-rank tests. To bound computation, GP kernel hyperparameters are estimated once per run (on the Protocol A training set) and reused across cross-validation folds. Template parameters are refitted within each fold from fold-training data only. Because the Protocol A training sample spatially overlaps the Protocol B test blocks, this reuse could in principle leak information favoring the GP-based methods. A control in which kernel hyperparameters were refitted within each fold from fold-training data only yielded indistinguishable results (physics-guided GP MAE 0.189±0.048 vs. 0.188±0.047; median $R^{2}$ 0.61 in both cases; per-run MAE differences ≤0.005, in both directions), so the shortcut has no material effect. Uncertainty calibration under Protocol B is assessed by the empirical coverage of ±1σ and ±2σ intervals and by the Spearman correlation between predicted σ and absolute error. All results are reported as mean ± standard deviation (SD) over the seven runs. No run or seed is excluded. The experimental comparison additionally includes random forest (RF) and inverse-distance weighting (IDW) baselines, together with the physics-guided GP. RF and IDW are reported only for the experiments (being tangential to the smooth-prior-versus-MLP mechanism the simulation isolates), whereas the multi-kernel weighted GP and Poisson kriging are evaluated under both simulation and experiment.

## 3. Results

### 3.1. Simulation: Uniform Sampling Favors the MLP

**Figure 4:**
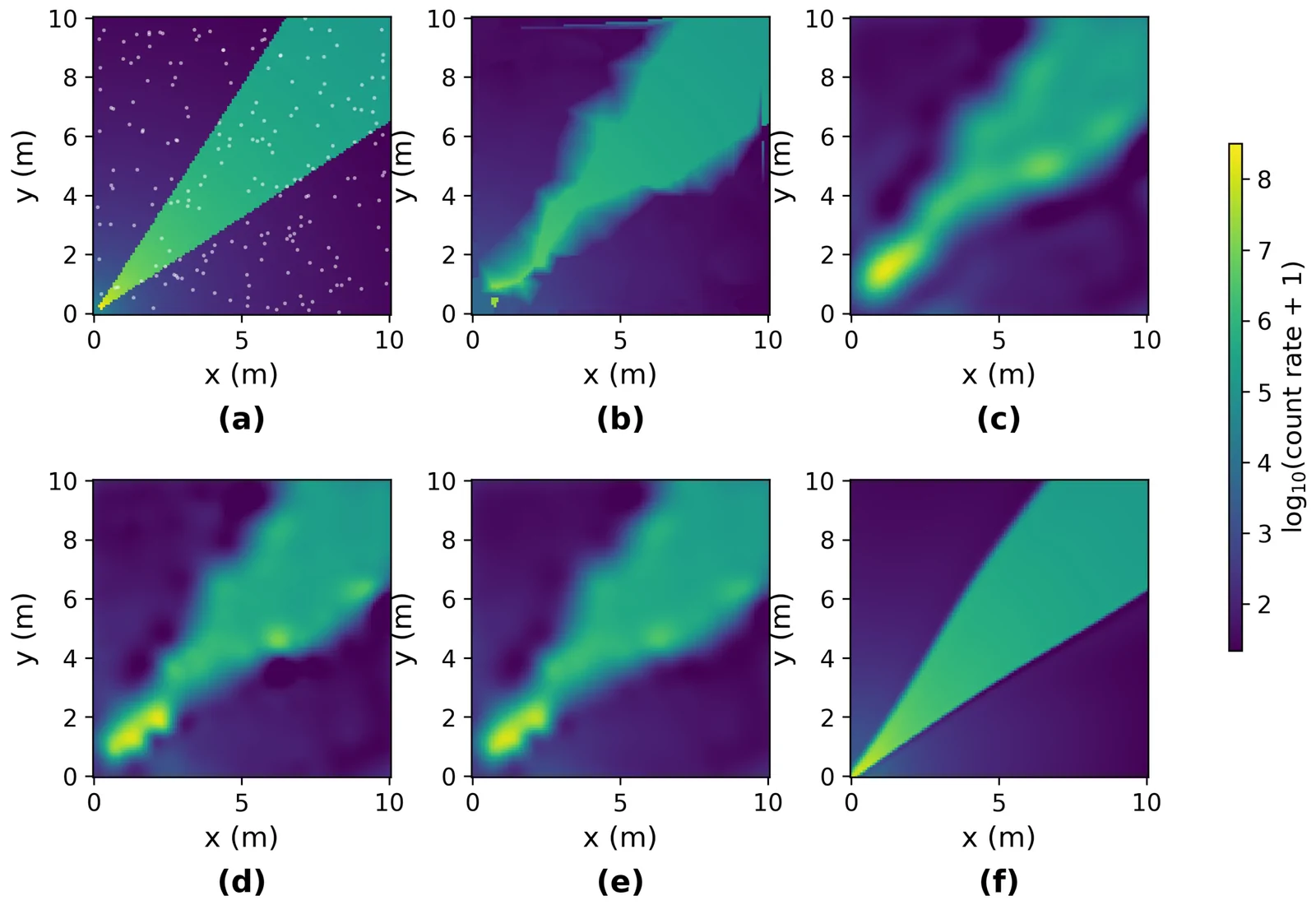
Simulation of the collimated source with Poisson-sampled counts: (**a**) ground truth field with sample locations (white dots); (**b**) linear interpolation; (**c**) GP with RBF kernel; (**d**) GP with Matérn 3/2 kernel and bias term (equivalent to ordinary kriging); (**e**) Poisson kriging; (**f**) MLP reconstruction. The smooth-prior methods (**b**–**e**) blur the beam edge, whereas the MLP (**f**) reproduces the sharp sector; all panels share the color scale on the right.

Table 1 summarizes truth-referenced errors under Poisson noise (five data seeds). The MLP dominates: MAE 0.096±0.014 versus 0.31–0.46 for all other methods, $R^{2}$ 0.947±0.014 versus 0.81–0.84. Figure 4 shows why: smooth-prior methods blur the beam edge, while the MLP reproduces the sector shape nearly exactly. This advantage is not an artifact of favorable initialization: across 18 runs spanning three data seeds and six network seeds, MLP $R^{2}$ remained within 0.932–0.966, never overlapping the GP range (0.808–0.850). Under uniform, well-covering samples, the absence of a smoothness prior is an asset, and MLP instability is negligible.

### 3.2. Protocol A: Method Parity at Visited Locations

At held-out points along the trajectory (Table 2), the ranking differs from the simulation: linear interpolation and random forest attain the best point metrics ($R^{2}$ 0.904 and 0.909), with inverse-distance weighting close behind (MAE 0.100, $R^{2}$ 0.875), and the MLP holds no advantage over the plain GP (MAE 0.112 vs. 0.108; all paired differences p≥0.078). The physics-guided GP matches the plain GP ($R^{2}$ 0.884), as expected, since, in near-dense data, the residual process dominates the template. Boundary F1 is likewise similar across all nine methods (0.859–0.905; best: linear interpolation 0.905±0.038; physics-guided GP 0.868±0.069). Seed sensitivity of the MLP correlates with data volume: the across-seed standard deviation of $R^{2}$ was ≤0.012 in the six denser runs but 0.069 (range 0.48–0.69) in the sparsest run (521 points). Random splits therefore measure neighborhood interpolation, for which most competent methods suffice.

### 3.3. Protocol B: The Physics-Guided GP Performs Best in Extrapolation

**Figure 5:**
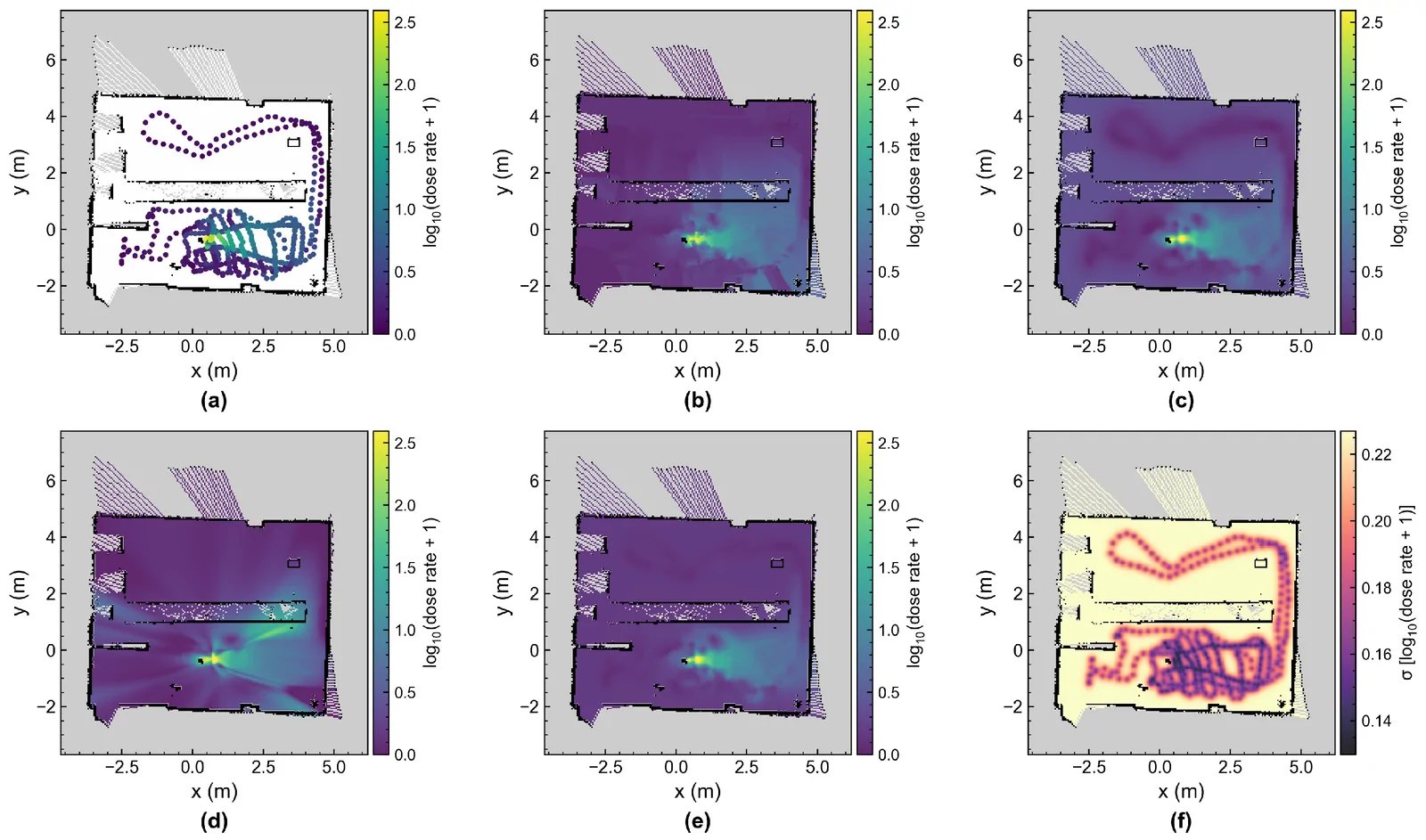
Experimental run 3 (the run with the median physics-guided-GP extrapolation MAE, chosen as representative), reconstructions overlaid on the SLAM occupancy-grid map (metric coordinates): (**a**) measurements at the survey locations; (**b**) linear interpolation; (**c**) plain GP (Matérn 3/2 with bias term); (**d**) MLP; (**e**) physics-guided GP; (**f**) predictive standard deviation of the physics-guided GP. Each reconstructed field is masked by the room map so that only the surveyed free space is colored, while walls and unmapped cells show the underlying gray map. Note the MLP streak artifacts far from the trajectory in (**d**) and the low-σ corridor along visited paths in (**f**).

**Figure 6:**
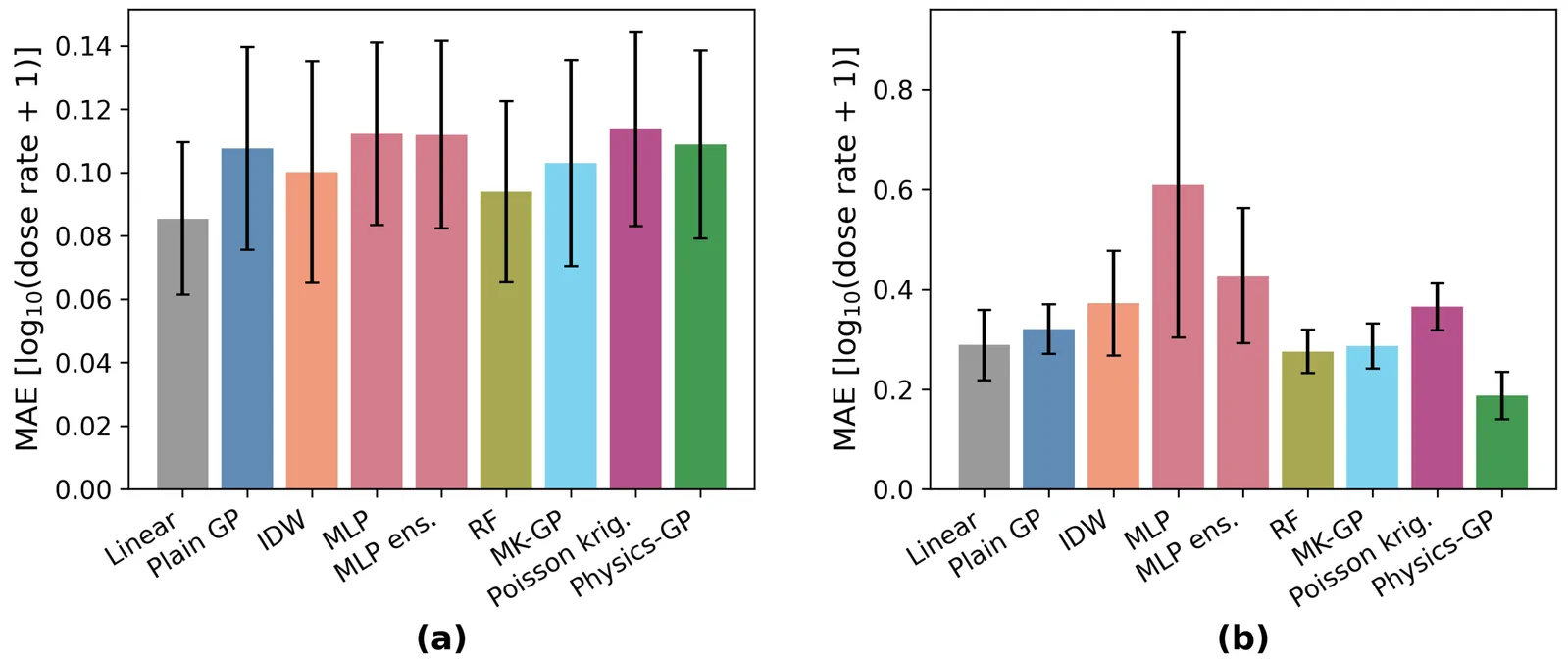
MAE of nine methods under (**a**) random hold-out (interpolation) and (**b**) spatial block cross-validation (extrapolation), mean ± SD over 7 runs.

Spatial block cross-validation reverses the simulation ranking (Table 3). Every purely data-driven method degrades severely in unvisited regions; the MLP degrades the worst (MAE 0.609±0.305; median $R^{2}=-5.7$), exhibiting the streak artifacts visible in Figure 5, and seed-ensembling mitigates but does not repair it (MAE 0.427). The physics-guided GP attains the highest median extrapolation $R^{2}$ (0.61, interquartile range (IQR) [0.51,0.69]; mean 0.565±0.198, identical to variant M0 of the ablation in Table 4), well above the strongest baseline, the multi-kernel weighted GP (0.31), and achieves an MAE of 0.188±0.047: 35% below linear interpolation, 35% below the multi-kernel weighted GP, 41% below the plain GP, 32% below random forest, 49% below Poisson kriging, 50% below IDW, and 69% below the single MLP. The advantage is consistent over every baseline on all three metrics: the physics-guided GP had the lower error in all seven runs against each of the eight baselines (7/7), which is the most extreme outcome the two-sided paired Wilcoxon signed-rank test can return at n=7 (minimum attainable p=0.016). Because this floor exceeds even the first-step Holm–Bonferroni threshold across the eight per-baseline comparisons (0.05/8=0.00625), no single comparison remains nominally significant after correction at n=7; the evidential weight of these results therefore rests on the magnitude of the error reductions (32–69%) and their directional consistency across every run and every baseline, not on nominal significance (see Section 4). Boundary F1 in unvisited regions is 0.694±0.134 for the physics-guided GP versus 0.43–0.61 for all baselines (Table 3), with the higher per-run F1 against every baseline in at least six of the seven runs. Figure 6 juxtaposes the two protocols and makes the reversal visible at a glance: moving from random hold-out to spatial blocks inflates the mean MAE of every purely data-driven method by a factor of 2.8–5.4 (linear interpolation 3.4×, random forest 2.9×, multi-kernel weighted GP 2.8×, plain GP 3.0×, Poisson kriging 3.2×, IDW 3.7×, single MLP 5.4×), whereas the physics-guided GP degrades by only 1.7×, the smallest factor among all methods.

### 3.4. Source Parameters Recovery and Calibrated Uncertainty

**Figure 7:**
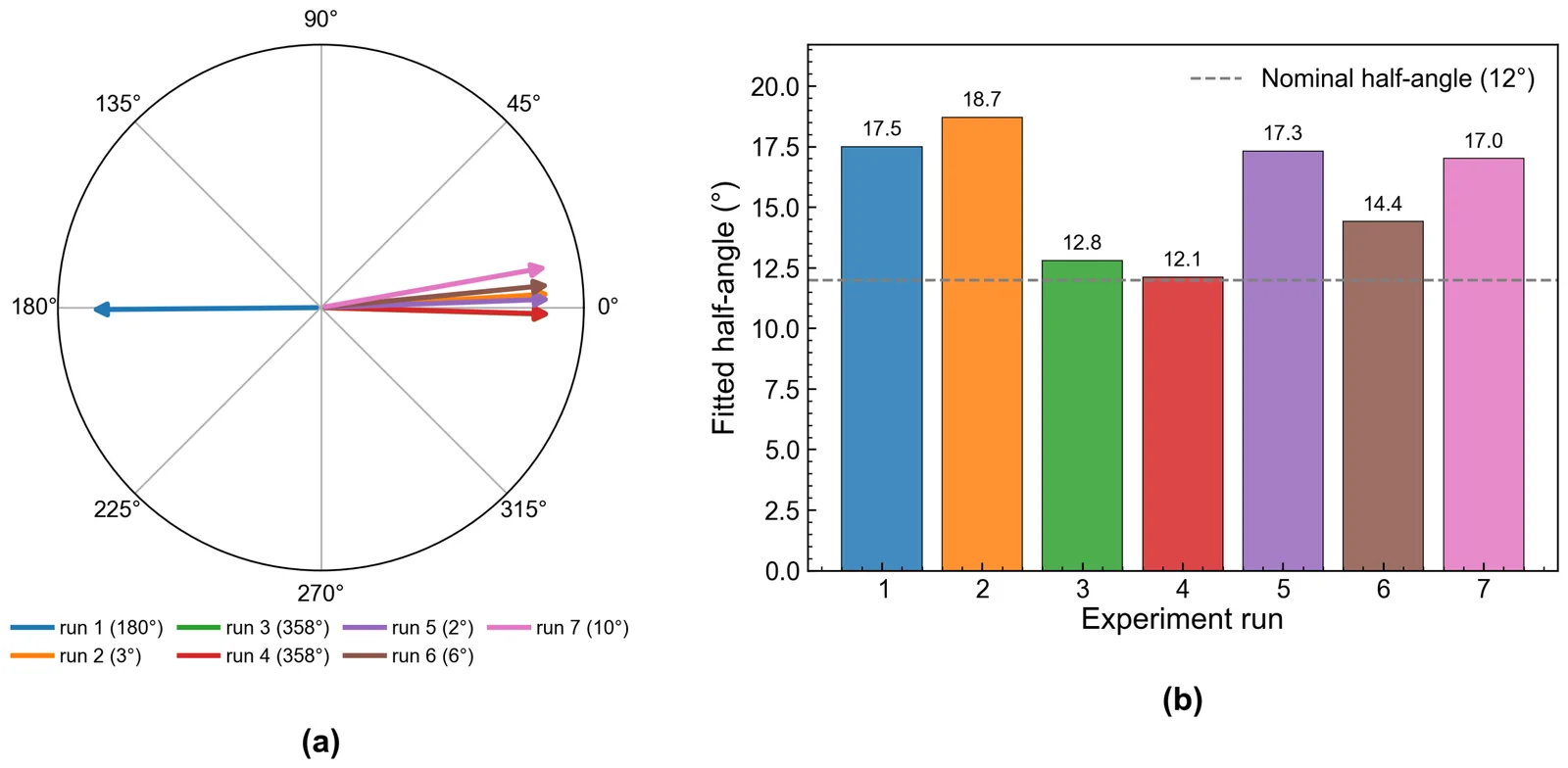
Recovered source parameters across the seven runs: (**a**) fitted beam direction, shown as a unit arrow per run on a polar axis (the true beam points toward 0°); (**b**) fitted collimator half-angle, with the dashed line marking the nominal value (12°). Six runs agree within ±10° of the true direction; run 1 is the mirror-flipped (180°) degenerate solution discussed in the text, while its half-angle remains in the common range.

**Figure 8:**
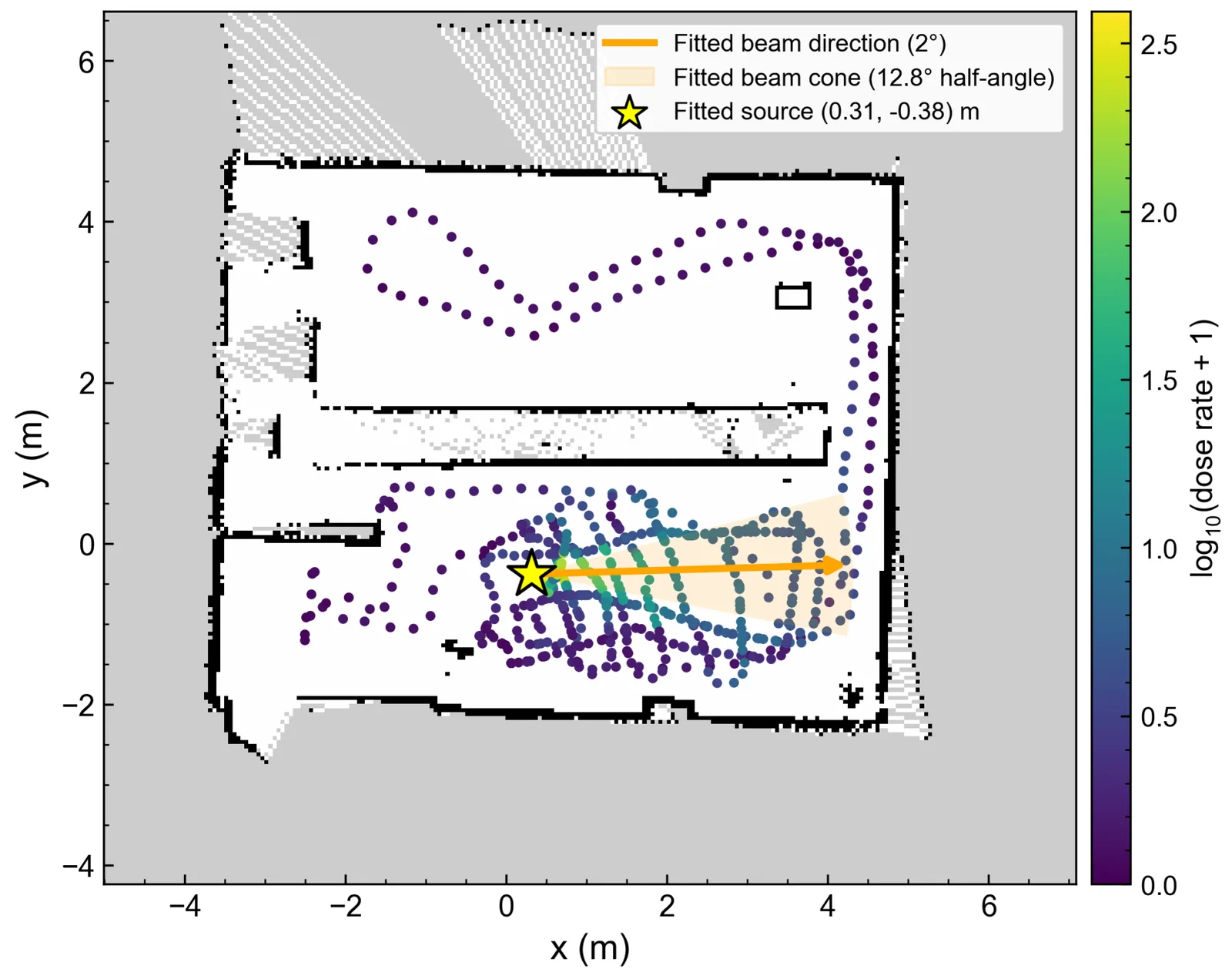
Source recovery for a representative run (run 3), overlaid on the SLAM map: measurement positions colored by log dose rate, the fitted source (star), the fitted beam direction (arrow), and the fitted beam cone (shaded, 12.8° half-angle). The recovered source sits at the leading edge of the high-dose measurements, and the fitted cone encloses the observed dose lobe, which decays with distance along the beam axis.

The template parameters, fitted independently per run, are mutually consistent and physically plausible (Figure 7). For six of the seven runs, the fitted beam direction falls within −2° to +10° of the true source orientation, and the fitted half-angle spans 12–19°. Figure 8 illustrates a representative fit (run 3) overlaid on the survey map: the recovered source lies at the leading edge of the high-dose measurements and the fitted beam cone encloses the observed dose lobe, confirming that the recovered geometry is consistent with the raw data. That the fitted half-angles cluster above rather than around the certificate value of 12° is physically expected: the finite penumbra of a real collimator and residual scattering off surrounding structures broaden the apparent beam beyond its geometric aperture, so an intensity-based fit should recover an effective half-angle slightly larger than the nominal one. The nominal 12° therefore acts as a lower bound on the fitted values, consistent with what is observed.

Run 1 is the sole exception: its fitted direction is the mirror image of the true beam (180.5°, plotted as −179.5° in Figure 7), while its half-angle (17.5°) remains in the same range as the other runs. This is a symmetry-induced local minimum rather than a fit failure: when the fitted source position lies near the centroid of the trajectory, a beam and its 180°-reversed counterpart explain the trajectory-sampled intensities almost equally well, and the least-squares objective admits both as near-degenerate solutions. The magnitude parameters (source position and half-angle) are unaffected by this sign flip; the run’s reconstruction accuracy is on par with the others (Table 2 and Table 3), and the multi-start initialization, which seeds directions rotated by ±90° and 180°, resolved the ambiguity in every cross-validation fold. The single-run whole-data fit shown here retains the mirrored solution only because it reports the global least-squares optimum without the fold-level restarts. The mapping thus yields a by-product of operational value: an estimate of source position, beam direction, and aperture without any dedicated inversion step, with the one known degeneracy identified and controlled.

**Figure 9:**
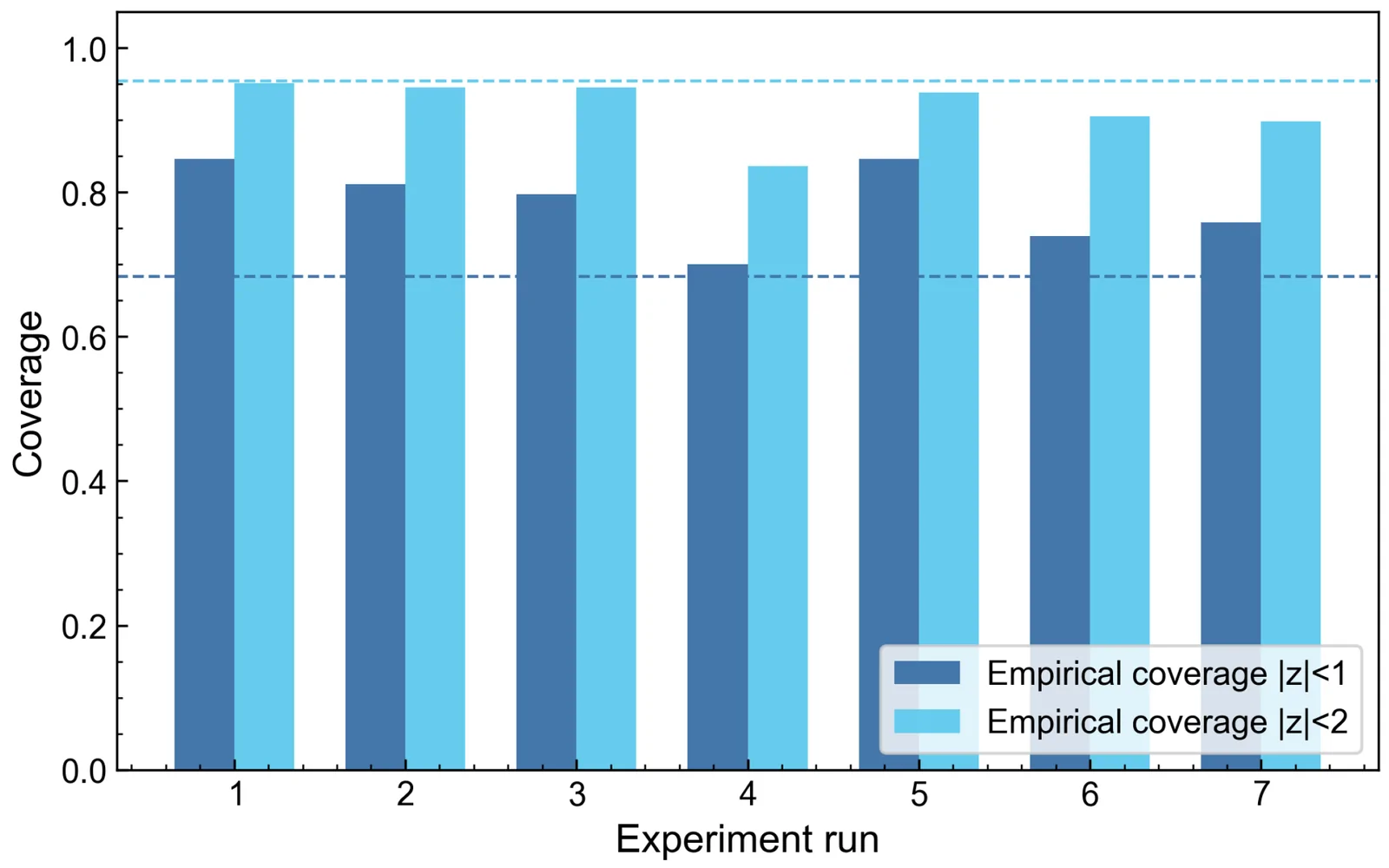
Empirical coverage of ±1σ and ±2σ prediction intervals of the physics-guided GP under spatial block cross-validation; dashed lines mark nominal Gaussian coverage.

Uncertainty calibration under Protocol B (Figure 9) shows empirical coverage of 0.70–0.85 for ±1σ (nominal 0.683) and 0.84–0.95 for ±2σ (nominal 0.954). The intervals are slightly overconfident in the worst run but remain usable for confidence mapping. Point-wise discrimination is weaker: the Spearman correlation between predicted σ and absolute error ranged from −0.58 to +0.06, reflecting the stationary kernel’s distance-driven variance. The predictive-σ map in Figure 5 illustrates the practical value nonetheless: uncertainty is low along the trajectory and high in unvisited regions, exactly the information a survey planner needs to direct supplementary measurements.

## 4. Discussion

### 4.1. Why the Rankings Differ

The simulation and experimental rankings are not contradictory; they are two regimes of one phenomenon. With uniform coverage, every location is near data, reconstruction is interpolation everywhere, and the MLP’s unconstrained flexibility wins at abrupt boundaries. With trajectory sampling, large areas are far from data and reconstruction is extrapolation: the MLP extrapolates without constraint (producing confident artifacts), a zero-mean GP shrinks toward a constant (blurring structure), linear interpolation clamps to nearest values (crude but bounded), and only a physically sensible mean function supplies the missing structure. Our preliminary tests indicated that an isotropic inverse-square prior offered only marginal improvement. This limited benefit is attributable to the anisotropic nature of the collimated radiation field, which is not adequately represented by an isotropic model. These findings underscore that a physics-based prior is effective only when it faithfully captures the underlying physical phenomenon.

### 4.2. Fairness of the Comparison

A potential objection is that the physics-guided GP wins the extrapolation comparison only because its mean function embeds the correct field family, whereas the purely data-driven baselines encode no structural prior. If any embedded template guaranteed the advantage, the comparison would be circular. To test this, we repeated the Protocol B evaluation with three deliberately altered mean functions, holding the residual-GP machinery and the spatial-block folds fixed (Table 4): an isotropic $1/r^{2}$ mean with no angular cutoff (M1); the same collimated template but with its beam direction forced 90° away from the fitted value, i.e., correct in structure but wrong in a key parameter (M2); and a constant mean, which reduces the method to the plain GP (M3). The correct template (M0) reproduces the reported result ($R^{2}=0.565$, MAE 0.188). Every misspecified mean collapses to negative $R^{2}$ and roughly doubles the MAE (M1: −1.43; M2: −1.98), landing near the no-prior lower bound (M3: −5.48). The advantage is therefore not conferred by embedding a template per se, but by embedding the *correct* one; a wrong prior is no better than none. Crucially, prior correctness is not privileged external knowledge but an observable quantity, and the fit-quality gate that exploits it can itself be quantified. Across all 35 spatial-block folds (7 runs × 5 folds), the misspecified means incur a normalized fit cost of at least 1.45× (M1) and 2.20× (M2) that of the correct template on the same fold. Using the leave-one-run-out threshold defined in Section 2.2 (τ=1.3× the highest correct-template cost among the remaining six runs), the gate detects 66 of 70 misspecified fits (94%) with 0 of 35 false alarms on correct fits. The four missed detections all occur in folds whose held-out blocks contain the beam core, so that the training data span only 65–80% of the run’s dynamic range and scarcely constrain any beam model. Detecting misspecification from such data is intrinsically hard, and this failure mode should be borne in mind operationally. Conversely, the absence of a warning must not be interpreted as proof that the template is adequate. With the gate in place, misspecification raises a runtime alarm rather than passing silently, prompting supplementary measurement or manual review; a conservative fallback to the plain GP (Section 2.3) is available as a default, though the stress test of Section 4 (Table 5) shows the freely fitted template is not worse than that fallback even under misspecification, so the gate’s value lies in flagging rather than in switching. Consistent with this, the physics-guided GP holds no advantage under Protocol A (Table 2), where dense data make the prior largely irrelevant. Its benefit is confined to the data-starved extrapolation regime where a correct prior is the only available source of structure.

### 4.3. Gate Robustness to Unseen Misspecification Types

**Figure 10:**
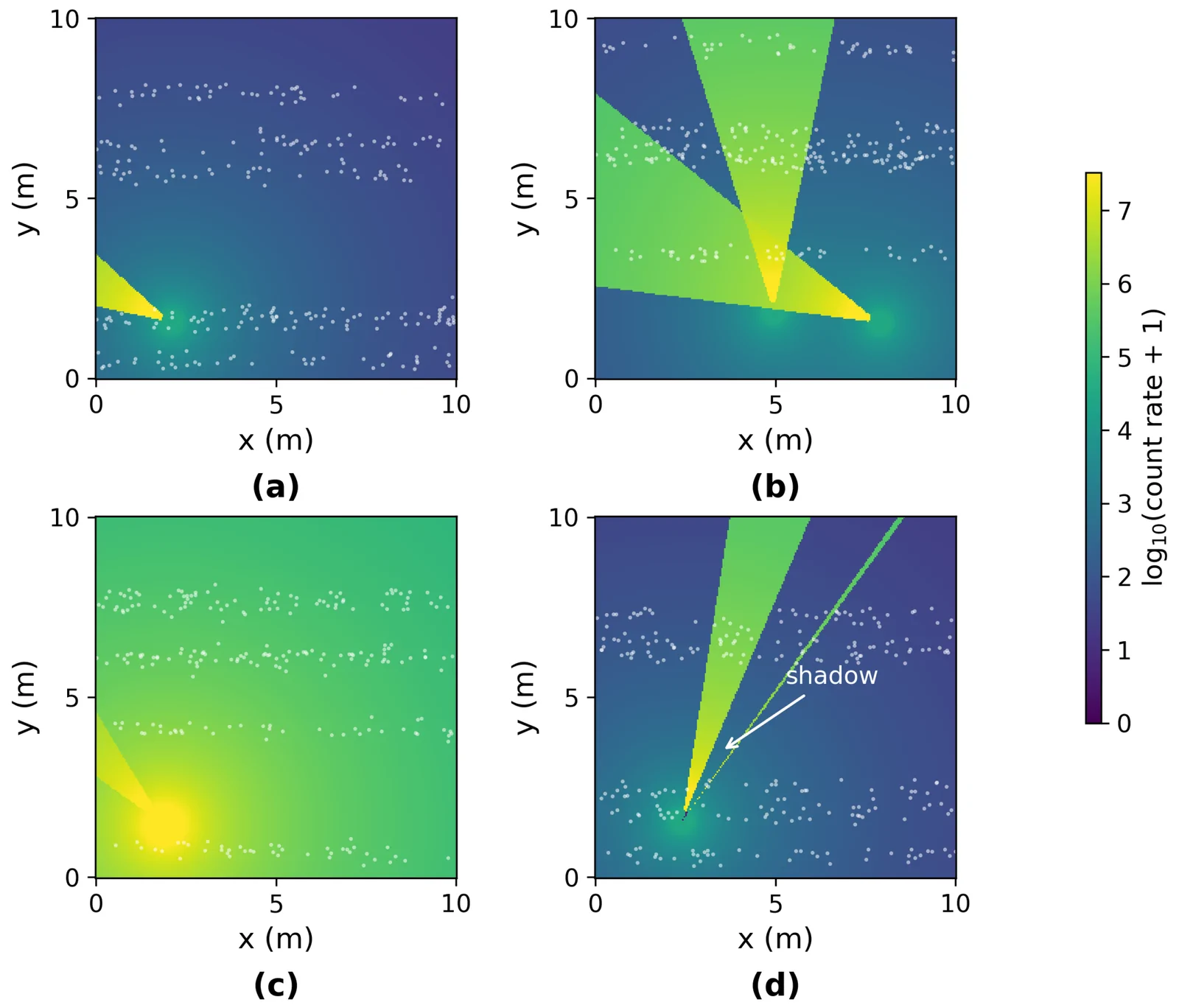
Simulated ground-truth fields for the gate stress test (log domain; white dots mark trajectory samples): (**a**) a correctly specified single collimated source; (**b**) multi-source superposition of two to three collimated beams; (**c**) a single beam overwhelmed by an isotropic scatter halo, which the template’s leakage and background terms absorb; (**d**) a single beam truncated by an occluding shadow (arrow), a localized anomaly that leaves the main lobe intact. Panels (**b**–**d**) violate the single-source template in structurally different ways.

The gate calibration above (94% detection, zero false alarms) is measured on the single-source experimental data with deliberately altered mean functions (M1, M2). Because no multi-source, scatter-dominated, or occluded fields were surveyed, we assess whether the gate generalizes to such qualitatively different misspecifications through a controlled simulation study. Using the same collimated-source physics as Section 2.3, we generated 22 correctly specified single-source fields and, as three unseen misspecification families, 20 realizations each of (i) multi-source superposition (two or three collimated sources), (ii) an isotropic scatter halo added to a single beam, and (iii) a single beam truncated by a wedge-shaped shadow (occlusion); Figure 10 shows a representative ground-truth field of each type. Each field was sampled along a trajectory-like path (six horizontal sweeps with unvisited bands between them) with Poisson counts; the single-source template was fitted, and its normalized fit cost $\bar{c}$ was recorded. Because the simulated fields differ from the experimental data in dynamic range and sampling density, their absolute $\bar{c}$ values are not comparable to the experimental threshold. We therefore re-calibrate the gate internally on the simulated single-source fields, using the same 1.3× leave-out rule, and report detection on the misspecified families (Table 5).

The gate flags 80% of multi-source fields, whose fit cost rises to roughly 80 times the correctly specified level, at a 9% false-alarm rate on held-out single-source fields, confirming that the mechanism transfers to a structurally distinct misspecification it was never calibrated against. Occlusion is detected less often (40%): a freely fitted single template still captures the visible lobe, so a localized shadow inflates the global cost only modestly, and this reduced sensitivity should be borne in mind operationally. The isotropic scatter halo is not flagged (0%), which is the correct behavior rather than a miss: the template’s leakage and background terms absorb the diffuse component, so the fit remains excellent (mean absolute error 0.052 versus 0.076 for the plain GP); the method tolerates scatter rather than being broken by it. Across all four families, the physics-guided GP reconstruction never exceeded the plain-GP fallback in mean error, so the gate is best read as an independent misspecification *alarm*, prompting operator caution or supplementary measurement, while the reconstruction itself degrades gracefully. A real multi-scenario validation remains necessary, but these results indicate the gate is not an artifact of the single dataset on which it was calibrated.

Because a global statistic can dilute a localized anomaly, we additionally evaluated a *spatial residual diagnostic*: within the spatial-block partition already used for cross-validation, each held-out block is scored by the mean standardized leave-block-out prediction residual ${\bar{z}}_{b}\sqrt{n_{b}}$, and a field is flagged when any block score exceeds a threshold calibrated on correctly specified fields by the same 1.3× leave-out rule. On an occlusion ensemble mirroring the experimental sweep geometry (20 occluded fields with 3–6° attenuating wedges; 22 correctly specified fields), the gate alone flagged 15 of 20, the block diagnostic 14 of 20, and their union 18 of 20 (90%), with zero false alarms; the diagnostic also names the affected blocks, localizing the anomaly spatially. Its limits mirror its logic: a shadow intersecting almost no survey samples is unlikely to be flagged by any residual-based check, and in the sparse random-trajectory regime of Table 5 wedges often fall between survey lines, where the diagnostic adds little. We therefore recommend reporting the block-level score map alongside the global gate whenever spatial block cross-validation is performed, at no additional model-fitting cost.

### 4.4. Sensitivity to Localization Error and Detector Smearing

Section 2.1 estimated that detector response time combined with platform motion smears an individual reading by approximately 0.4 m along the trajectory, and that SLAM contributes a localization error of 2.72±0.49 cm RMSE (measured in the motion-capture laboratory, since reference instrumentation cannot operate in the radiation laboratory). To quantify what these two effects do to boundary accuracy and recovered source parameters, we ran a paired simulation: twenty collimated-source realizations under the sweep-pattern trajectory sampling of Section 4.3, each evaluated (i) with ideal point measurements and (ii) with the measured perturbations injected, consisting of Gaussian position noise of 2.72 cm RMSE plus a 0.4 m boxcar average of the true rate along the trajectory before Poisson sampling. Table 6 reports the recovered-parameter errors. Under ideal sampling, the template recovery is essentially exact. With the perturbations injected, the recovery degrades gracefully in 17 of 20 realizations: the median source-position error rises to 0.08 m, the median beam-direction error to 0.26°, the fitted half-angle acquires a small negative bias (−0.18°), and, because the along-track smearing is symmetric about the beam edge, the recovered boundary position shifts by only about 0.01 m, an order of magnitude below the smearing scale itself; the smearing is absorbed almost entirely by the template’s transition-width parameter rather than displacing the boundary. The remaining 3 of 20 realizations produced gross misfits (two broad-cone solutions displaced from the true beam geometry and one half-angle collapse); all three raised the normalized fit cost to 35–48 times the ensemble median, so the fit-quality gate identifies them. These results support the claim that the ≈0.4 m smearing limits the reliable recovery of finer spatial features but does not materially bias the recovered geometry, and that the occasional catastrophic fit induced by smearing is observable rather than silent.

### 4.5. Relation to Prior Work

Read against the two traditions positioned in Section 1, our results sharpen that positioning rather than restate it. We deliberately retained the MLP as a strong, honestly reported baseline, and the simulation confirms its genuine superiority under dense uniform sampling. The collimation-aware prior helps only where trajectory sampling leaves large unvisited regions, and among the geostatistical methods it outperforms both plain GPR and the multi-kernel weighted GP in that regime (Table 3). The resulting recommendation is conditional, not partisan: choose by sampling geometry. Where coverage is guaranteed (e.g., gridded post-accident drone sweeps), neural interpolators excel; where surveys are trajectory-constrained, as in the common robotic case, the physics-guided GP is preferable, and its variance output can drive informative path planning [13,33] to convert extrapolation into interpolation where it matters. We do not include a U-Net radiation–reconstruction baseline [21]: such models require a large corpus of paired sparse-input/dense-truth fields for supervised training, whereas each survey here yields a single field (seven in total), a regime in which a U-Net cannot be trained or compared fairly. We therefore treat it as complementary prior work rather than a baseline under our data budget.

### 4.6. Template Selection and Adaptation

The method should be read as a two-layer construction. The outer layer, comprising a GP with a fitted parametric physics template as its mean, a residual process that supplies local correction and predictive variance, and the fit-quality gate that monitors template adequacy, is generic and contains nothing specific to collimation. The inner layer is the template family itself, and Equations (1) and (2) are one instantiation, appropriate to the single-dominant-collimated-source scenario validated here; any differentiable parametric field model (isotropic point source, line or plane source, superpositions of a few sources, or a template with an attenuating obstruction) can be substituted without altering the outer layer. For deployment in environments whose source configuration is not known in advance, template choice becomes a small model-selection problem. Each member of a candidate library is fitted, and selection follows a penalized fit criterion, for example, the normalized robust cost $\bar{c}$ already computed for the gate, or the GP marginal likelihood penalized for the number of template parameters as in the Bayesian information criterion. The plain GP is retained as the null member of the library, so that the framework degrades to ordinary kriging whenever no physical template is warranted. The fit-quality gate then serves double duty, ranking candidates during selection and flagging global misspecification at runtime. The multi-source row of the stress test (Table 5) illustrates the discriminative signal available for such selection: a wrong template incurs a fit cost roughly eighty times that of a correct one. Validating this selection loop on real multi-source and obstructed fields, and extending the template library to three-dimensional geometries, are the natural next steps.

### 4.7. Practical Implications

For ALARA-driven operations, the asymmetry of boundary errors argues for methods that expose their ignorance. The error level required of a map is set by the downstream decision rather than by a universal threshold. For work planning, the operative requirement is that the map assign each location to the correct dose-rate zone, where zone boundaries are conventionally spaced about one decade apart (e.g., the 10 µSv/h boundary threshold used for the boundary F1 metric). Under this requirement, the physics-guided GP’s unvisited-region MAE of 0.188 in the log domain corresponds to a typical dose-rate ratio of $10^{0.188}\approx 1.5$, well inside a one-decade zone width, whereas the baseline MAEs of 0.28–0.61 correspond to ratios of 1.9–4.1, large enough to misassign locations near zone boundaries; the boundary F1 of 0.69 versus 0.43–0.61 expresses the same margin in classification terms. Two qualifications apply: the boundary F1 is a classification score at the fixed 10 µSv/h threshold and does not quantify boundary-localization error in meters, and an operational acceptance threshold ultimately depends on application-specific dose action levels, instrument calibration and directional-response uncertainty, and independent reference measurements, which same-detector cross-validation cannot replace. Log-domain MAE ≲0.2, corresponding to typical errors below a factor of about 1.6 in dose rate, is therefore adequate for zone-level ALARA planning in fields of this dynamic range, whereas applications requiring dosimetric accuracy (e.g., stay-time calculation at a fixed workplace) still call for confirmatory in situ measurement. Two aspects of the uncertainty output must be distinguished. Its *interval coverage* is approximately calibrated (±2σ coverage 0.84–0.95), so aggregate confidence intervals can be used for regional risk flagging. Its *point-wise discrimination* is weak, however (Spearman σ–error correlation as low as −0.58 in the worst run), so predicted σ should not be read as a reliable per-point error ranking. In practice, the spatial σ map remains useful because its dominant signal is coverage-driven, low along visited paths and high in unvisited regions, which is precisely what identifies where supplementary measurements would be most informative. The recovered source parameters offer a sanity check against facility records and a seed for formal source inversion [34]. They should be used as such rather than as a standalone source confirmation: as the run-1 mirror-direction degeneracy shows, the beam direction can flip when the source lies near the trajectory centroid, so parameter recovery must be accompanied by the multi-start procedure and a fit-quality gate, and the direction in particular should be cross-checked before operational use.

Radiation counts follow Poisson statistics, so a Poisson-likelihood GP might be expected to fit better than the log-Gaussian model and to sharpen the weak point-wise uncertainty ranking noted above. We tested this directly. Because the detector reports an integrated ambient dose-rate rather than raw counts, a strict Poisson likelihood is not applicable to the experimental data; the comparison is run in simulation, where the counts are exactly Poisson. Over twelve collimated-source realizations under trajectory sampling, a Laplace-approximation Poisson GP with a log link (constant latent mean, Matérn 3/2 covariance) was compared against the log-Gaussian GP with the same kernel, both scored in the $\log_{10}\left(\mathrm{rate}+1\right)$ domain against the true field (Table 7). The log-Gaussian model had the lower reconstruction error in all twelve realizations (MAE 0.066±0.012 vs. 0.123±0.060; paired Wilcoxon p=0.0005) and a higher $R^{2}$, while the Poisson GP improved the σ–error rank correlation only marginally and not significantly (0.60±0.11 vs. 0.50±0.09; p=0.13), at comparable ±2σ coverage. Two effects explain this. First, the log transform serves to compress the several-decade dynamic range, not to stabilize Poisson variance; for pure Poisson counts the variance-stabilizing transform is a square root, and the residual heteroscedasticity of $\log_{10}\left(\mathrm{count}+1\right)$, largest at low counts, is absorbed by the white-noise kernel term. Second, the Laplace Poisson GP is less numerically stable under trajectory sampling, and its mode reverts to the mean rate in unvisited regions, offering no extrapolation advantage over the log-Gaussian mean. A full Poisson (or heteroscedastic) treatment therefore remains a reasonable option for uncertainty ranking, but yields no accuracy or calibration gain here and is not adopted.

### 4.8. Limitations

First, all experiments involve a single dominant collimated source in one indoor environment. Multi-source superposition, scatter-dominated fields, and non-line-of-sight geometries remain untested on real data, and the template family must be chosen to match the scenario (with fallback to the plain GP when the fit is poor). A simulation stress test (Table 5) indicates that the fit-quality gate detects multi-source misspecification reliably (80%) but is less sensitive to localized occlusion (40%), while correctly tolerating the simulated isotropic scatter halo; the block-level spatial residual diagnostic of Section 4.3 raises occlusion detection to 90% when the survey geometry samples the anomalous region, though no residual-based check can be expected to reliably detect a localized anomaly that is not sampled by the trajectory. A real multi-scenario validation nonetheless remains necessary. Second, n=7 runs limits statistical power: the paired Wilcoxon floor is p=0.016, and under a Holm–Bonferroni correction across the eight per-baseline comparisons none reaches α=0.05 (the n=7 floor p=0.016 exceeds even the first-step Holm threshold of 0.05/8=0.00625), so the evidential weight rests on the directional consistency (lower error in all seven runs against every baseline) rather than on nominal significance. Third, predicted σ ranks point-wise errors weakly. Heteroscedastic or non-stationary kernels are natural next steps, whereas a Laplace Poisson-likelihood GP, tested in simulation (Table 7), improved point-wise ranking only marginally and non-significantly while degrading reconstruction accuracy, so it is an optional rather than a clearly beneficial extension. Fourth, SLAM accuracy was evaluated in a motion-capture room rather than the radiation laboratory, and detector response combined with platform motion smears readings by ≈0.4 m, limiting the reliable recovery of finer spatial features; the paired-simulation sensitivity analysis of Section 4.4 indicates that these perturbations bias the recovered boundary by only ≈0.01 m and the source parameters modestly (median position error 0.08 m, direction 0.26°) in 17 of 20 realizations, with the remaining gross misfits flagged by the fit-quality gate. Finally, the direction ambiguity observed in run 1 indicates that template fitting should in practice be accompanied by the multi-start procedure and the fit-quality gate quantified above (94% detection, zero false alarms on this dataset), whose known blind spot, training data that exclude the beam core, should be respected when interpreting gated results. Because both the gate and the block-level residual diagnostic depend on whether the trajectory samples the anomalous region, coupling the calibrated posterior uncertainty to informative path planning, building on the uncertainty- and information-driven exploration strategies developed for gas distribution mapping [14] and for safe robot exploration under GP prediction [33], is the most direct route to removing that dependence; extending the framework to cooperative multi-robot surveys [8] would additionally test whether the anisotropic prior transfers across platforms sharing one field.

## 5. Conclusions

We investigated reconstruction of strong-gradient radiation fields from mobile robot surveys, with dual evaluation protocols separating interpolation from extrapolation, and reached three conclusions. (1) For the single dominant collimated source studied here, sampling geometry, rather than algorithm choice alone, determines method ranking: a coordinate-input MLP is robustly the best boundary reconstructor under uniform sampling (simulation $R^{2}$ 0.95, stable across 18 seed combinations), yet the worst extrapolator under trajectory sampling. (2) Under spatial block cross-validation on held-out measurements, the proposed physics-guided GP, embedding a fitted collimated-beam template as the GP mean, achieved the highest median extrapolation $R^{2}$ (0.61; best baseline 0.31), reduced unvisited-region MAE by 32–69% relative to all eight baselines (lower error in all 7 runs against every baseline), delineated the beam boundary most accurately (F1 0.69), and recovered source position, direction, and aperture, with the direction consistent with the apparatus in six of seven runs and all seven half-angles in a plausible range (one known mirror degeneracy). (3) Its predictive uncertainty was approximately calibrated at the region level (±2σ coverage 0.84–0.95) and maps directly onto operational needs, flagging untrustworthy regions and prioritizing supplementary measurements, thereby supporting the precise, decision-ready boundary delineation that field operations and emergency response require, although predicted σ does not reliably rank individual point errors and should be used for region-level confidence only. Future work will extend the template family to multiple and unknown source types, exploit the variance output for active survey planning, deepen uncertainty calibration, and generalize to three-dimensional fields.

## Figures and Tables

**Table 1:** Simulation, truth-referenced errors (mean ± SD over 5 seeds; log domain). The best result in each column is shown in bold.

Method	MAE	RMSE	$R^{2}$
MLP	0.096±0.014	0.426±0.053	0.947±0.014
Linear	0.313±0.020	0.773±0.054	0.827±0.025
Multi-kernel weighted GP	0.355±0.019	0.741±0.026	0.842±0.011
Poisson kriging	0.386±0.015	0.769±0.028	0.829±0.012
GP (Matérn 3/2 + bias)	0.387±0.012	0.765±0.035	0.831±0.015
GP (RBF)	0.461±0.020	0.813±0.047	0.809±0.023

**Table 2:** Protocol A, random hold-out (mean ± SD over 7 runs; log domain). The best result in each column is shown in bold. Values are cross-validation estimates against held-out measurements from the same detector system; no independent dense experimental ground-truth map was measured.

Method	MAE	RMSE	$R^{2}$	Boundary F1
Linear	0.085±0.024	0.167±0.049	0.904±0.061	0.905±0.038
Random forest	0.094±0.029	0.163±0.047	0.909±0.062	0.880±0.060
IDW	0.100±0.035	0.183±0.067	0.875±0.107	0.876±0.058
Multi-kernel weighted GP	0.103±0.033	0.176±0.057	0.887±0.093	0.859±0.079
Plain GP	0.108±0.032	0.180±0.057	0.885±0.094	0.865±0.067
Physics-guided GP	0.109±0.030	0.183±0.052	0.884±0.083	0.868±0.069
MLP	0.112±0.029	0.188±0.052	0.877±0.092	0.863±0.069
MLP ensemble (3 seeds)	0.112±0.030	0.188±0.055	0.876±0.098	0.861±0.071
Poisson kriging	0.114±0.031	0.188±0.047	0.879±0.083	0.870±0.059

**Table 3:** Protocol B, spatial block cross-validation (over 7 runs; log domain). The best result in each column is shown in bold. These are held-out cross-validation estimates rather than errors against an independently measured dense experimental ground truth. MAE and RMSE are mean ± SD; $R^{2}$ is reported as median [IQR] because isolated cross-validation folds with near-zero test-block variance drive the $R^{2}$ mean and SD of the purely data-driven methods to large, uninformative negative values (e.g., one fold in run 4 yields $R^{2}\approx -2900$, inflating the single-MLP $R^{2}$ mean to −86±200 over the seven runs); MAE and RMSE, being bounded, are unaffected and reported as mean ± SD.

Method	MAE	RMSE	$R^{2}$ (Median [IQR])	Boundary F1
Physics-guided GP	0.188±0.047	0.277±0.051	0.61[0.51,0.69]	0.694±0.134
Random forest	0.276±0.043	0.393±0.056	0.18[0.04,0.39]	0.471±0.151
Multi-kernel weighted GP	0.287±0.045	0.353±0.054	0.31[0.13,0.44]	0.612±0.119
Linear	0.289±0.070	0.385±0.081	0.24[0.03,0.41]	0.486±0.132
Plain GP	0.320±0.049	0.387±0.063	0.10[−1.25,0.27]	0.518±0.122
Poisson kriging	0.365±0.046	0.443±0.059	−0.09[−1.76,0.01]	0.428±0.133
IDW	0.373±0.105	0.500±0.092	−0.12[−1.06,0.04]	0.584±0.154
MLP ensemble	0.427±0.135	0.553±0.151	−0.83[−3.30,−0.11]	0.587±0.075
MLP	0.609±0.305	0.789±0.348	−5.66[−9.00,−1.26]	0.529±0.164

**Table 4:** Mean-function ablation under Protocol B (spatial block cross-validation; mean ± SD over 7 runs, log domain). The best result in each column is shown in bold. Only the GP mean function is changed; the residual process and folds are identical. M0 is the method as reported.

Mean Function	MAE	RMSE	$R^{2}$
M0: collimated template (correct)	0.188±0.049	0.276±0.053	0.565±0.198
M1: isotropic $1/r^{2}$ (no angular cutoff)	0.434±0.065	0.523±0.063	−1.43±1.67
M2: template, beam direction 90° off	0.443±0.068	0.528±0.060	−1.98±2.25
M3: constant mean (=plain GP)	0.460±0.051	0.536±0.056	−5.48±8.26

**Table 5:** Fit-quality gate on unseen misspecification types (simulation; trajectory sampling, log domain). $\bar{c}$ is the normalized template fit cost; detection and false-alarm rates use a threshold re-calibrated within the simulation (not comparable to the experimental 94% figure). Reconstruction MAE compares the (possibly misspecified) physics-guided GP against the plain-GP fallback.

Field Type	$\bar{c}$ (Median)	Gate Flags	Phys.-GP MAE	Plain-GP MAE
Single source (correct)	0.0016	9% (false alarm)	0.102	0.300
Multi-source (2–3)	0.137	**80%**	0.411	0.552
Occlusion (shadow wedge)	0.012	40%	0.141	0.349
Scatter-dominated (halo)	≈0	0% (absorbed)	0.052	0.076

**Table 6:** Sensitivity of template-parameter recovery to measured perturbations (simulation; 20 paired collimated-source realizations, sweep-pattern trajectory sampling; median [IQR]). The perturbed condition applies Gaussian pose noise of 2.72 cm RMSE plus a 0.4 m along-track boxcar smearing of the rate before Poisson sampling. The perturbed column summarizes 17 of 20 well-behaved realizations; the 3 gross misfits (normalized fit cost 35–48× the ensemble median) are flagged by the fit-quality gate and excluded from the summary statistics.

Recovered Quantity (Error)	Ideal Sampling	Perturbed Sampling
Source position (m)	0.001[0.001,0.003]	0.079[0.055,0.088]
Beam direction (°)	0.01[0.00,0.01]	0.26[0.06,0.34]
Half-angle bias (°)	−0.00[−0.01,+0.01]	−0.18[−0.30,−0.07]
Boundary displacement (m)	0.000[0.000,0.000]	0.012[0.010,0.013]

**Table 7:** Poisson-likelihood GP versus the log-Gaussian GP (simulation; twelve collimated-source realizations, trajectory sampling; scored in the $\log_{10}\left(\mathrm{rate}+1\right)$ domain against truth). ρ is the Spearman correlation between predicted σ and absolute error. The best result in each column is shown in bold.

Method	MAE	$R^{2}$	ρ(σ,∣err∣)	±2σ cov.
Log-Gaussian GP (this work)	0.066±0.012	0.33±0.10	0.50±0.09	0.97
Poisson GP (Laplace)	0.123±0.060	−0.15±0.74	0.60±0.11	0.96

## Data Availability

The evaluation framework, the physics-guided GP implementation, all baselines (including the multi-kernel weighted GP and Poisson kriging), the fit-quality-gate stress-test and Poisson-GP comparison scripts, the seven survey runs, and all per-run and per-realization results are openly available at https://github.com/Hubery-Lee/PhysicsGuidedGP-RadiationMapping (accessed on 16 August 2026) (MIT-licensed).
